# Targeting Monoacylglycerol Lipase in Pursuit of Therapies for Neurological and Neurodegenerative Diseases

**DOI:** 10.3390/molecules26185668

**Published:** 2021-09-18

**Authors:** Anca Zanfirescu, Anca Ungurianu, Dragos Paul Mihai, Denise Radulescu, George Mihai Nitulescu

**Affiliations:** Faculty of Pharmacy, “Carol Davila” University of Medicine and Pharmacy, Traian Vuia 6, 020956 Bucharest, Romania; anca.zanfirescu@umfcd.ro (A.Z.); dragos_mihai@drd.umfcd.ro (D.P.M.); denise.radulescu@stud.umfcd.ro (D.R.); george.nitulescu@umfcd.ro (G.M.N.)

**Keywords:** monoacylglycerol-lipase inhibitors, 2-arachidonoylglycerol, neuroinflammation, neurodegenerative disorders, carbamates, irreversible inhibitors, benzisothiazolinone, piperidine, pyrrolidone, azetidinyl amides

## Abstract

Neurological and neurodegenerative diseases are debilitating conditions, and frequently lack an effective treatment. Monoacylglycerol lipase (MAGL) is a key enzyme involved in the metabolism of 2-AG (2-arachidonoylglycerol), a neuroprotective endocannabinoid intimately linked to the generation of pro- and anti-inflammatory molecules. Consequently, synthesizing selective MAGL inhibitors has become a focus point in drug design and development. The purpose of this review was to summarize the diverse synthetic scaffolds of MAGL inhibitors concerning their potency, mechanisms of action and potential therapeutic applications, focusing on the results of studies published in the past five years. The main irreversible inhibitors identified were derivatives of hexafluoroisopropyl alcohol carbamates, glycol carbamates, azetidone triazole ureas and benzisothiazolinone, whereas the most promising reversible inhibitors were derivatives of salicylketoxime, piperidine, pyrrolidone and azetidinyl amides. We reviewed the results of in-depth chemical, mechanistic and computational studies on MAGL inhibitors, in addition to the results of in vitro findings concerning selectivity and potency of inhibitors, using the half maximal inhibitory concentration (IC_50_) as an indicator of their effect on MAGL. Further, for highlighting the potential usefulness of highly selective and effective inhibitors, we examined the preclinical in vivo reports regarding the promising therapeutic applications of MAGL pharmacological inhibition.

## 1. Introduction

Monoacylglycerol lipase (MAGL; EC 3.1.1.23) is a membrane-bound α/β serine hydrolase, which selectively metabolizes 2-monoacylglycerols [1]. Its main substrate is 2-arachidonoylglycerol (2-AG), an endogenous agonist of the cannabinoid receptors 1 and 2 (CB1R and CB2R) and a precursor of arachidonic acid (AA), and, consequently, of downstream-derived eicosanoids, thus interfering with the eicosanoid signaling [2]. 2-AG can be metabolized by multiple enzymes; however, MAGL is the main enzyme involved in its metabolism, regulating its hydrolysis rate into AA and glycerol [2].

**Figure 1:**
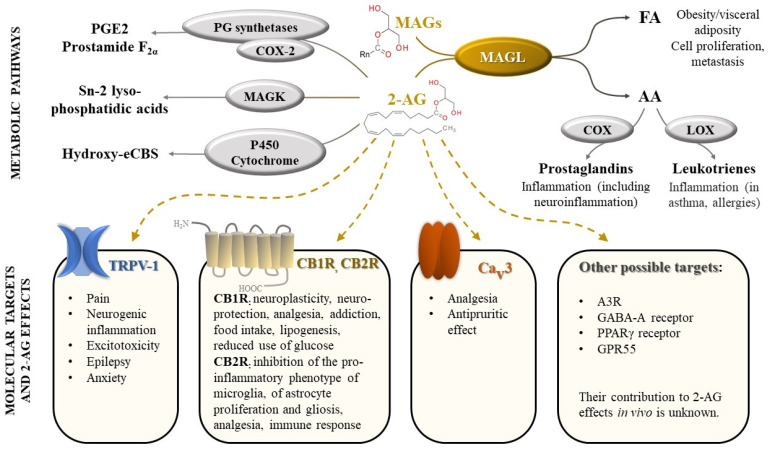
Monoacylglycerol lipase (MAGL), an enzyme with central role in a complex biological network, regulating cannabinoid signaling and arachidonic acid metabolism. Legend: FA-fatty acids; AA-arachidonic acid; COX—cyclooxygenase; LOX—lipoxygenase; MAGs—monoacylglycerols (*n* = 8–18); 2-AG—2-arachidonoylglycerol; PG—prostaglandin; PGE2—prostaglandin E2; MAGK—monoacylglycerol kinase; eCBs—endocannabinoids; TRPV-1—transient receptor potential vanilloid type 1; CB1R—cannabinoid receptors 1; CB2R—cannabinoid receptors 2; Ca_v_3 T-type calcium channel; A3R—adenosine receptor 3; GABA-A receptors- γ-Aminobutyric acid type A receptors; PPAR-γ receptors—Peroxisome proliferator-activated γ receptors; GPR55—G protein-coupled receptor 55.

2-AG is one of the most abundant endocannabinoids and plays an essential role in the regulation of many physiological processes. It modulates neuroplasticity and neuroprotection by mediating retrograde suppression of inhibitory or excitatory currents in GABAergic and glutamatergic synapses [3] by activating CB1R receptors [4]. Furthermore, CB1R receptor activation by 2-AG is associated with modulation of several other neurotransmissions: dopaminergic, noradrenergic, serotonergic, with consequences on behavior (e.g., food intake, addiction), memory and pain perception [1,5]. 2-AG appears to also regulate metabolism, cell proliferation, pain sensation, and reproductive and cardiovascular functions [6,7], by activating CB2R receptors and other targets (peroxisome proliferator-activated receptors, transient receptor potential vanilloid-1) [7] (Figure 1).

Activation of immune cells (e.g., macrophages) leads to an increase in 2-AG production, which, in turn, decreases pro-inflammatory mediators and re-establishes homeostasis [8]. However, the main metabolite of 2-AG, AA, is well known for its involvement in inflammation, among other physiological processes, acting as a substrate for several enzymes such as cyclooxygenases (COX-1 and COX-2), thus being converted into pro-inflammatory prostaglandins (PGs) and thromboxanes [8,9]. MAGL appears to be the main enzyme involved in AA synthesis in the brain, liver, and lung [10,11].

In addition to 2-AG, MAGL hydrolyzes other monoacylglycerides, affecting the levels of free fatty acids, which later serve as a precursor pool for pro-tumorigenic signaling lipids [1].

Therefore, the inhibition of MAGL appears to be a promising therapeutic target for various pathologies, such as neurodegenerative and neuropsychiatric diseases, chronic pain or cancer [1,12]. In recent years, a plethora of MAGL inhibitors were synthetized, often containing reactive carbamate or urea moieties, resulting in an irreversible inactivation of the enzyme by covalent modification of the active serine residue, an interaction well-described in previously published reviews [1,13].

The main advantage of covalent inhibition is that it offers a sustained pharmacological response [14]. Some recent identified irreversible inhibitors present a good pharmacokinetic profile and are highly effective in reducing inflammation in vivo [15,16]. However, chronic administration of irreversible inhibitors was associated with negative in vivo effects, such as CB1R receptor downregulation and physical dependence. Therefore, the focus of recent studies was the development of reversible inhibitors, lacking these unwanted effects and with an optimal pharmacokinetic profile.

In this review, we aim to revise the latest data regarding MAGL inhibitors, focusing on their most important structural features. We highlight the results of in-depth chemical, mechanistic and computational studies on MAGL inhibitors, and the most promising therapeutic uses reported in recent in vivo studies, focusing on papers published between 2015 and 2021—a period of intense research in this emerging field.

## 2. Molecular Characterization of MAGL and Mechanism of Catalysis

Biologically organized as a dimer, this 33 kDa enzyme possesses three main structural features: a catalytic triad, a nucleophilic elbow, and an oxyanion hole. The core consists of a β-sheet with seven parallel and one antiparallel strands, surrounded by six α-helices, with the active site formed by three amino acid residues: Ser122, Asp239 and His269, which are located between β-strands and α-helices of the α/β-hydrolase fold [17,18], at the bottom of the long channel under the lid domain of the enzyme [19].

The serine residue (Ser) is placed in the conserved sequence Gly-His-Ser-Met-Gly, representative of α/β-serine hydrolases, also known as “a nucleophilic elbow”, which basically forms a pocket, connecting to the exterior via a connection channel. The core is covered by a highly variable domain—a cap [13]. Inside the cap domain and lining the active site access, a highly lipophilic α-helix allows anchoring of the enzyme to the cell membrane, thus making it possible for the protein to recruit its lipophilic substrates. Several hydrophobic residues cover the connection channel, interacting with the arachidonoyl moiety of 2-AG and ensuring substrate specificity [12,20]. This flexible hydrophobic tunnel becomes more polar towards the catalytic site, allowing the accommodation of the glycerol group. Another narrow tunnel, perpendicular to the connection channel, connects the catalytic site with the outside of the protein and is coated by Pro178, Ser181, Leu184, Tyr194, Arg202 and Ala203, allowing the glycerol moiety, released after substrate hydrolysis, to exit [12].

MAGL selectively hydrolyses 2-monoacylglycerols with different fatty acid chain lengths (from C8:0 to C18:0) and saturation. MAGL preferentially hydrolyses unsaturated compared to saturated substrates (e.g., arachidonoyl glycerol vs. palmitoyl glycerol) [12]. It does not cleave diacylglycerols, triacylglycerols [21], cholesterol esters or prostaglandin glycerol esters [22].

The MAGL catalytic mechanism consists of two different phases: the binding of the substrate and the hydrolysis of the ester bond [12], and involves two main reactions: acylation and diacylation. Catalysis starts by an acylation [23]: once the substrate reaches the active site, Ser122 triggers a nucleophilic attack to the carbonyl of the substrate, forming a complex with it [16]. This attack is facilitated by an acid-base mechanism in which Ser122 is activated by a proton transfer that starts when Asp239 deprotonates His269, which takes another proton from the hydroxyl group of Ser122 [12]. Consequently, the hydroxyl residue of the serine, with subsequently increased nucleophilicity, attacks the carbonyl group of the substrate [23]. The tetrahedral intermediate formed during the transition state is placed in a cavity of the enzyme named the “oxyanion hole” [16]. The oxyanion hole stabilizes the charge distribution and reduces the state energy of the tetrahedral intermediate by forming at least two hydrogen bonds [23]. The release of glycerol as the leaving group from the tetrahedral intermediate leads to an acyl-enzyme complex [12]. Then, the diacylation step takes place with the nucleophilic attack of an activated water molecule to the carbonyl group of the acylated complex, releasing AA and regenerating the enzyme [12,23].

## 3. Development of Pharmacophore Models for MAGL Inhibitors

To date, the usage of computer-aided drug design (CADD) tools has been widely preferred in the rational drug discovery of MAGL inhibitors, with several research groups employing CADD approaches on several chemical scaffolds to discover and identify novel MAGL inhibitors. Afzal et al. proposed the 3D QSAR pharmacophore model for MAGL inhibitors using a ligand-based pharmacophore generation [24]. Accordingly, the pharmacophore comprised three features: one hydrogen bond acceptor, one positive center (e.g., piperazine) and three aromatic rings. The same group further investigated the structural features needed to inhibit MAGL. Using hybrid ligand and structure-based approaches, they established three key essential features for MAGL inhibition: an amphiphilic benzene-fused heterocyclic ring (to form a π–π stacking interaction with Tyr194 and also interact with the polar and non-polar amino acids) linked with a bulky hydrophobic fragment (to fill up the hydrophobic space and to interact with the hydrophobic amino acids) connected through a linker having a carbonyl functional group, either in cyclic or acyclic amide form, or in a heterocyclic system (to form an H-bond with one or more key amino acid residues, Ala51, Met123 and Ser122 of the oxyanion hole in the active site) [25].

Examination of the MAGL X-ray structure, combined with the analysis of the structural elements of diphenylmethane inhibitors, led to the development of a novel 3D pharmacophore model, focused on the correct distance and reciprocal orientation of the aromatic rings within the inhibitor structure. The key feature is the longer distance between the centroids of the aromatic systems (~7 Å). The spatial arrangement of the aryl moieties, constrained by a dihedral angle of 119.4°, is critical to maximize the interaction with the hydrophobic pockets of the enzyme, and provides the correct accommodation of the molecule within the catalytic site [26].

Jha and co-workers (2021) developed a pharmacophore-based virtual screening (VS) protocol aimed at the identification of new reversible MAGL inhibitors, which was based on the latest X-ray structure of MAGL in a complex with a reversible non-covalent inhibitor [27]. Accordingly, the pharmacophore comprises five mandatory features (four hydrogen-bond acceptors and a hydrophobic feature—one aromatic ring group) and three optional hydrophobic features (a chlorine, a terminal aromatic ring group and a thiazole ring). This pharmacophore model provided high predictability for estimating the activities of a variety of compounds. When employing all eight features, the virtual screening results provided 5707 new hits from amongst 276,150 molecules, thereby proving that the model is highly selective. Moreover, the authors highlighted the importance of the structural water as an indirect anchoring point to MAGL binding site [28].

From the analysis of available data concerning MAGL reversible inhibition, the key pharmacophoric elements that appear to be important for inhibitory activity are: (a) a carbonyl group able to interact in the oxyanion hole of the enzyme, (b) an aromatic portion also comprising a heterocyclic nucleus involved in van der Waals interactions in a closed hydrophobic region of the binding site, and (c) a second aromatic portion, which interacts in the opening [29].

## 4. MAGL Inhibitors

During the past decade, various irreversible and reversible MAGL inhibitors were discovered. Although the first-generation inhibitors were capable of blocking MAGL in in vitro pharmacological assays and showed beneficial effects in preclinical tests, their lack of selectivity led to various side effects, such as CB1R desensitization, tolerance and physical dependence. Nevertheless, the structure optimization of these inhibitors (e.g., insertion of hexafluoroisopropanol as a leaving group) inspired the further development of selective MAGL inhibitors, with a better safety profile [13,30].

In this review, we focus on presenting the compounds designed during the past five years, and derivatives with proven high selectivity for MAGL, rather than other enzymes involved in endocannabinoid processing, which hold promise for optimal pharmacokinetics and improved efficacy in disorders, such as neurodegenerative diseases, chronic pain and chronic inflammatory diseases. Although we used the half maximal inhibitory concentration (IC_50_) as an indicator of each compound’s effect on MAGL, one should consider that this parameter is only a relative indicator of the inhibitor efficacy because testing protocols often differ [31].

### 4.1. Covalent Irreversible Inhibitors

**Figure 2:**
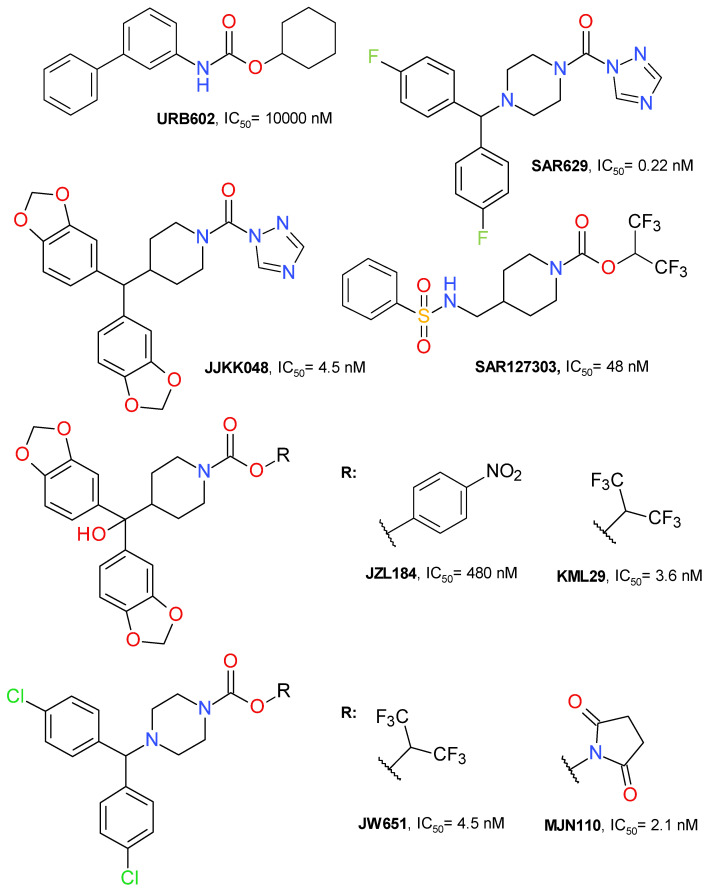
Representative irreversible MAGL inhibitors.

Irreversible well-characterized inhibitors (Figure 2) contain an electrophilic moiety which inactivates the enzyme by bonding covalently, typically to the reactive side chain of an amino acid in the active site [1]. The electrophilic fragment is often called a warhead in medicinal chemistry and its choice can significantly reduce the toxicity and increase the selectivity towards similar targets [32]. The most used warheads in the development of MAGL covalent inhibitors are carbamate, urea, maleimide or oxadiazolone [1].

The first selective MAGL inhibitor identified was JZL184, a 4-nitrophenyl carbamate derivative. KML29 is an analogue of JZL184 with hexafluoroisopropyl alcohol (HFIP) as the leaving group. This change improved the inhibitory potency and reduced the off-target effects. JW651 is also a HFIP carbamate and is structurally similar to KML29. SAR127303 is a highly potent HFIP piperidine carbamate inhibitor of MAGL. MJN110 is the N-hydroxysuccinimidyl (NHS) analogue of JW651 with a superior potency than KML29 [33,34]. The chemical structures of all these irreversible inhibitors are depicted in Figure 2.

The inhibitors must be highly lipophilic in order to interact with the target MAGL binding pocket, which is correlated with poor water solubility. This is a challenge for further development and leads to safety risks due to increased metabolic clearance/off-target activities [1]. Newer derivatives, reviewed in this paper, are designed by carefully selecting the warhead and by establishing an equilibrium between lipophilicity and hydrophilicity for an optimal pharmacokinetic profile.

A clear, simple classification of the MAGL inhibitors is difficult to establish. Most irreversible inhibitors are based on the carbamate scaffold. One criterion for classification may be the nature of the alcohol bounded by the carbamate unity. The research on the mechanism by which carbamates inhibit MAGL highlighted the importance of a low pKa value for the alcohol; HFIP is the most used.

HFIP has a pKa of 9.3 and its structure is similar to that of the glycerol moiety of AG, the natural MAGL substrate. HFIP carbamate derivatives have good aqueous chemical stability over a pH range of 2 to 8. HFIP is released after MAGL’s carbamoylation and its safety profile is supported by the clinical experience with sevoflurane [35]. Administered as a volatile anesthetic, sevoflurane is mainly metabolized to fluoride and HFIP, which is rapidly glucuronidated and excreted as HFIP-glucuronide in urine [36].

In general, the HFIP carbamate offers a high selectivity towards serine hydrolases, but there are some common off-targets that include the serine hydrolases fatty acid amide hydrolase (FAAH), α/β hydrolase domain containing 6 (ABHD6), phospholipase A2 group VII (PLA2G7), and the carboxyesterases (CES) [37]. ABHD6 is connected to the endocannabinoid signaling by its capacity to hydrolyze 1-arachidonoyl-glycerol (1-AG) and 2-AG [38].

Another important scaffold, although not used as often as the carbamate template, is represented by the urea derivatives. SAR629 (Figure 2) is a derivative of urea substituted with piperazine and triazole that inhibits MAGL at nanomolar concentrations by forming a carbamylated adduct through Ser122 residue [12,39]. JJKK048 (Figure 2) is also a triazole urea derivative with a structure that is highly similar to those of carbamate derivatives JLZ184 and KLM29. It potently inhibits MAGL with an IC_50_ value of 0.36 nM and is selective in rapport with FAAH and ABHD6 [39]. Not all triazole ureas inhibit MAGL in an irreversible manner [40].

#### 4.1.1. Azetidine HFIP Carbamates

**Figure 3:**
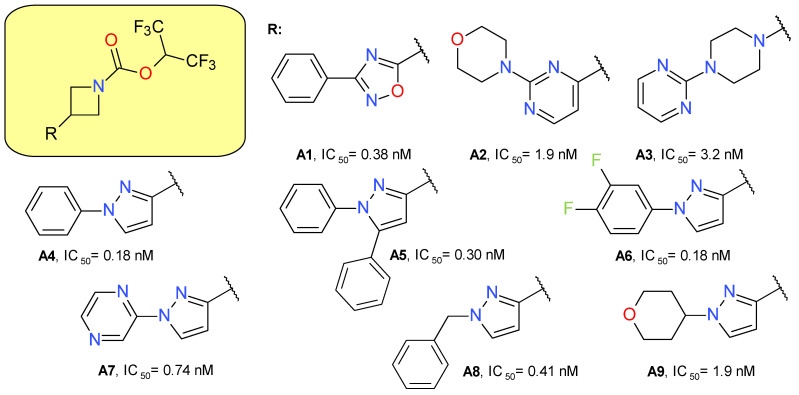
Irreversible MAGL inhibitors—azetidine carbamate derivatives.

Butler et al. (2017) used azetidine as a linker between the HFIP carbamate moiety and the lipophilic scaffold [14]. A secondary heterocyclic ring was added directly to the azetidine ring, in which the substitution in the 3 position was essential to provide a linear configuration. All the used heterocycles—oxadiazole, pyrimidine, piperazine and pyrazole—provided potent MAGL inhibitors, but the latter was chosen for development (Figure 3). The use of the pyrazole ring to link the active electrophilic group with a hydrophilic tail led to an increased inhibitory potency and optimized ligand efficiency and fit quality values [14].

The pyrazole derivative **A4** was tested at both 1 and 10 µM, showing significant inhibition against ABHD6, CES 1 and 2, and PLA2G7. The inhibitory effects on FAAH were observed only for the 10 µM concentration. 

The administration of compound **A4** in mice (10 mg/kg subcutaneously) increased 2-AG brain concentration up to five-fold and the effect duration was an average of 8 h. The major disadvantage of the compound was the rapid clearance, leading the research group to hypothesize that ring cores larger than azetidine will increase the stability of the inhibitor and of its adduct with MAGL. The piperidine analog of **A4** proved to have a prolonged pharmacodynamic effect [14].

#### 4.1.2. Piperazine HFIP Carbamates

The piperazine HFIP carbamate scaffold was previously used to develop JW651, a benzhydrylpiperazine derivative that potently inhibits MAGL with an IC_50_ of 38 nM, with no significant effect on other brain serine hydrolases at concentrations up to 10 μM. Oral administration in C57Bl/6J mice at 5 mg/kg augmented several-fold the brain levels of 2-AG and reduced accordingly the AA levels without affecting the *N*-arachidonoylethanolamide (AEA) concentration [37].

**Figure 4:**
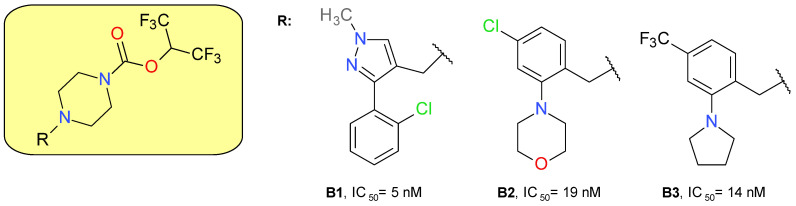
Irreversible MAGL inhibitors—piperazine carbamate derivatives.

Cisar et al. (2018) designed and synthesized various biaryl- and monoaryl-methyl analogues of JW651 as potent MAGL inhibitors. The substituted pyrazolylmethyl piperazines derivatives presented a potent inhibitory effect on MAGL and favorable lipophilicity, but a low selectivity towards ABHD6 and PLA2G7. The structure **B1** represents the most potent MAGL inhibitor of these series (Figure 4). The replacement of the pyrazolylmethyl fragment with benzyl maintained MAGL inhibitory potency but reduced the selectivity towards ABHD6. The introduction of a morpholine fragment (**B2**) improved the selectivity profile. The pyrrolidine benzyl derivative **B3** demonstrated both high potency and selectivity for MAGL. Compounds **B2** and **B3** completely inhibited MAGL action in the brain of mice administered a 5 mg/kg dose by oral gavage [35].

A mass spectrometry-based assay on **B3**, named ABX-1431, and recently Lu AG06466, confirmed the irreversible inhibition of MAGL by the carbamoylation of the catalytic Ser122 residue [35]. **B3** is a lipophilic basic amine molecule, but its activity on the hERG channel is weak (IC20 = 7 μM) [41]. 

#### 4.1.3. Azabicyclo[3.1.0]Hexane Trifluoromethyl Glycol Carbamates

**Figure 5:**
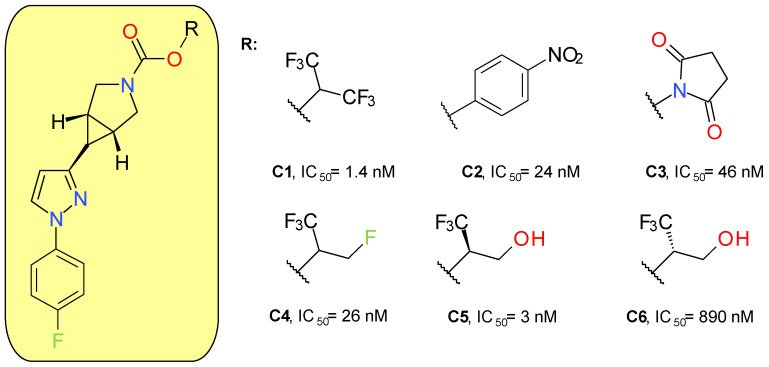
Irreversible MAGL inhibitors—azabicyclo[3.1.0]hexane trifluoromethyl glycol carbamates and related structures.

McAllister et al. (2018) developed a new series of MAGL inhibitors based on the pyrazole azetidine scaffold of compounds such as **A4** by replacing the rapidly hydrolysable azetidine with azabicyclo[3.1.0]hexane. In order to optimize the compounds’ solubility and their pharmacokinetic parameters, various types of carbamates were attached to the bicyclic ring (Figure 5). Compound **C1**, a HFIP carbamate, demonstrated the best inhibitory potency on MAGL and a very good selectivity in rapport with FAAH, but a very high lipophilic character and a low solubility. The analog *p*-nitrophenol carbamate (**C2**) produced a potent inhibitory effect on both MAGL and FAAH [42]. The low selectivity of **C2** towards FAAH confirms previous results on *p*-nitrophenol carbamates such as JZL184 [43]. The use of NHS carbamate (**C3**) decreased the inhibitory potency but provided very good selectivity on MAGL compared to FAAH, CES 1 and 2, and ABHD6.

Applying small changes to HFIP provided compounds with better pharmacokinetic profiles. The authors underlined that branching in the leaving group pocket is an important structural feature for maintaining selectivity compared to other enzymes involved in endocannabinoid processing. Compounds **C5** and **C6** were designed to better mimic 2-AG, the natural enzyme substrate. The racemic mixture of **C5** and **C6** demonstrated very good potency (IC_50_ = 6 nM) and selectivity. The major difference between the activities of the two enantiomers indicates that, in addition to carbamate reactivity, the binding interactions between the enzyme and the leaving group are also important factors [42].

The lead compound of this series (**C5**) was named PF-06795071 and was assayed on a panel of related serine hydrolases displaying no significant effects, with the exception of CES 1 (80% inhibition at 10 μM). Following intravenous administration in rats and dogs, **C5** displayed a short half-life of approximately 1 to 2 h [42]. A ^18^F-labeled analogue of **C5** was synthesized and administered to Sprague Dawley rats, demonstrating a good brain uptake and distribution [44].

#### 4.1.4. Azetidone Triazole Ureas

**Figure 6:**
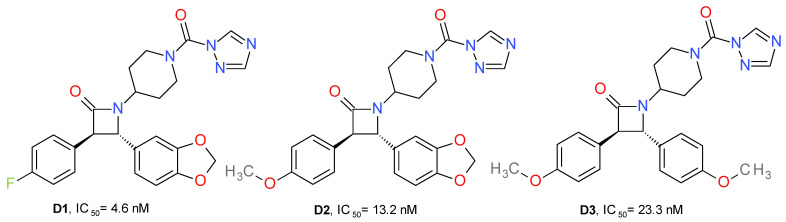
Irreversible MAGL inhibitors—*trans*-3,4-diarylsubstituted *β*-lactam derivatives.

A series of new urea MAGL inhibitors were developed using as a template JJKK048 and SAR629. The *trans*-3,4-diarylsubstituted *β*-lactam structural motif was used to obtain an optimal configuration for MAGL inhibition (Figure 6). 4-Fluorophenyl and the methylene-3,4-dioxyphenyl moieties provide the key hydrophobic interactions with the enzyme, which is reflected in an increased inhibition potency of human MAGL. Compound **D1** presented the best potency on MAGL and close to 900-fold selectivity compared to FAAH. Its enantiomer, the 3*S*, 4*R* isomer, displayed eight-fold lower potency on MAGL and less selectivity than FAAH. The exchange of the fluorine atom with a methoxy group (**D2**, **D3**) slightly reduced the MAGL potency and significantly reduced the selectivity towards FAAH [26].

Potency is also related to the pKa of the leaving group’s conjugated acid. Accordingly, the triazole moiety offered the best pharmacokinetic profile (pKa = 10). This can be observed because the imidazole analogue of **D1** presented no significant inhibitory effects on MAGL. Similarly, the HFIP and *p*-nitrophenyl carbamates analogues of **D1** had lower potencies [26].

#### 4.1.5. Benzisothiazolinone Derivatives

In the structure of MAGL there are three important cysteine residues—Cys242, Cys201 and Cys208—that regulate the enzyme’s catalytic activity. Cys242 is situated near the catalytic Ser122 and represents a target of several covalent inhibitors of MAGL. The group of maleimide derivatives irreversibly inhibit MAGL by forming a covalent S-alkylated adduct through a Michael addition reaction. N-arachidonoyl maleimide has a medium inhibitory effect on MAGL, with an IC_50_ value near 1 μM [30]. Disulfiram and several related disulfide derivatives also target the Cys residues of MAGL, but the mechanism consists of the formation of disulfide bonds [45].

**Figure 7:**
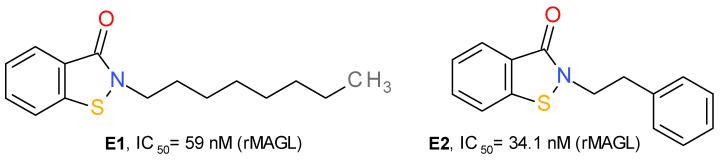
Irreversible MAGL inhibitors—benzisothiazolinone derivatives.

Based on the structure of octhilinone and octylbenzisothiazolinone (**E1**), two potent irreversible inhibitors of MAGL with IC_50_ values of 88 and 20 nM, respectively [13], Castelli et al. (2020) designed a series of N-substituted benzisothiazolinone derivatives. Compound **E2** (Figure 7) potently inhibits the activity of MAGL. This effect was significantly lower on MAGL mutated at various Cys residues. The authors demonstrated that **E2** forms a reducible disulfide bond with Cys201, and not Cys242. Structurally related compounds irreversibly inhibited the bacterial sortase A enzyme by the same mechanism, i.e., the formation of a disulfide bond with the catalytic Cys residue [13].

### 4.2. Reversible Inhibitors

The first reported reversible MAGL inhibitors were euphol and pristimerin; however, the two terpenoids lack selectivity, and thus were not considered as potential scaffolds for further development of MAGL inhibitors [46].

The first developed reversible, non-competitive MAGL inhibitors, benzo[d][1,3]dioxol-5-ylmethyl 6-([1,1′-biphenyl]-4-yl)-hexanoate and (4-(4-chlorobenzoyl)piperidin-1-yl)(3-hydroxyphenyl)methanone, were effective in vivo (they improved the clinical outcome of multiple sclerosis in a mouse model of autoimmune encephalomyelitis [47] and relieved the neuropathic hypersensitivity induced by oxaliplatin [48], respectively), with no impairment of motor and cognitive functions, thus confirming the hypothesis that reversible MAGL inhibitors possess a better safety profile than irreversible inhibitors [47,48].

#### 4.2.1. Salicylketoxime Derivatives

**Figure 8:**
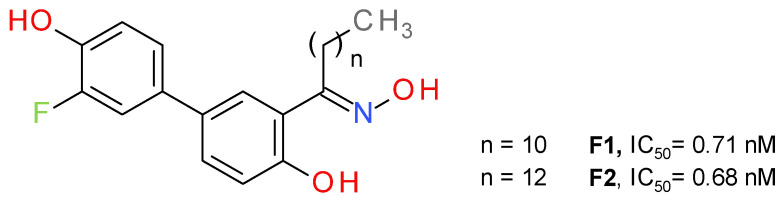
Reversible MAGL inhibitors—salicylketoxime derivatives.

Bononi et al. a used salicylketoxime scaffold, with a peripheral phenolic ring substituted with a fluorine atom in a position *ortho* to the hydroxyl group [46]. The potency of inhibition increased in direct proportion with the number of methylene units in the alkyl group of the ketoxime moiety, up to nanomolar inhibition values (Figure 8). The highest inhibitory activity was reported for the compounds with a linear saturated ketoximic alkyl chain, which can be explained by the structural similarity to the endogenous substrate 2-AG. Inserting simple aromatic groups in the ketoxime moiety (e.g., phenyl ring or a benzyl group) decreased the inhibitory effect. The most active compounds, **F1** and **F2**, were shown to possess good antiproliferative potency against cancer cells, particularly in ovarian and breast cancer [46].

#### 4.2.2. Piperidine Derivatives

Piperidine derivatives possessing an amidic carbonyl group are promising scaffolds for the development of reversible MAGL inhibitors. This moiety forms two H-bonds with the nitrogen backbone of Ala51 and Met123. The piperidyl is directed toward an open cavity of the protein and interacts with Leu148, Leu213 and Leu241 [49]. Various derivatives using this scaffold were identified and their inhibitory efficacy was assessed.

**Figure 9:**
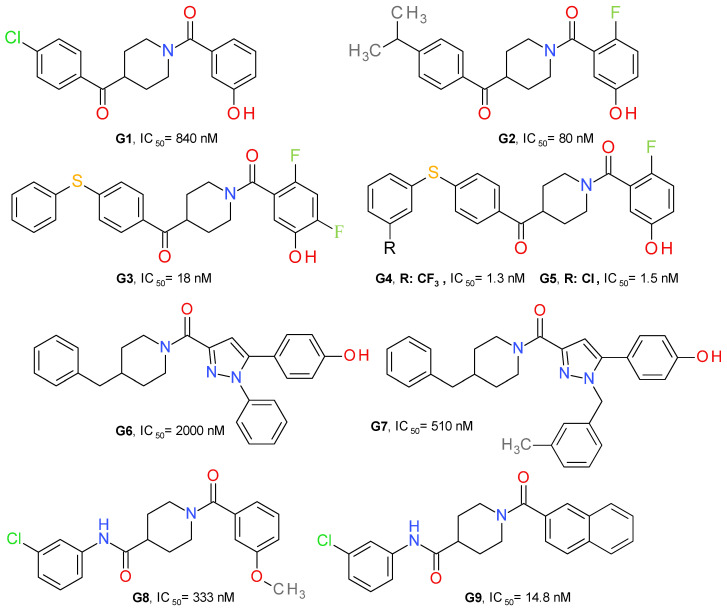
Reversible MAGL inhibitors—piperidine derivatives.

Granchi et al. (2016) identified 1-benzoylpiperidine derivatives as candidates for developing selective MAGL inhibitors. The first identified inhibitor was a piperidine molecule substituted in position 4 with 4-chlorobenzoyl and with 4-methoxybenyoyl at its N-atom. The replacement of the piperidine central ring with a piperazine abolished the effect on MAGL. The substituent in position 4 was maintained and several modifications at the N-benzoyl scaffold resulted in 4-(4-chlorobenzoyl)piperidin-1-yl)(4-hydroxyphenyl)methanone (**G1**), with an IC_50_ of 840 nM (Figure 9). It possessed reversible interaction properties and antiproliferative activity in cancer cells [50].

Further development of more potent inhibitors starting from the above-mentioned lead, was reported. A fluorine atom in *para* to the phenolic hydroxyl group on the amidic phenyl ring or to the amide carbonyl group appears to be beneficial for MAGL inhibition potency, such as compound **G2**. The methoxylated analog of **G2** displayed a higher IC_50_ value (2600 nM) compared to **G2**, thus confirming that the hydroxyl group is essential for the interaction with MAGL. The docking studies demonstrated the role of the hydroxyl group by bonding to Glu53 and His272 [51].

The class of 1-benzoylpiperidine-based MAGL inhibitors was expanded and several structure-activity relationships were deduced. The exchange of the fluorine atom in **G2** with other groups, such as triflouromethyl, nitro or amino, proved to be detrimental for the MAGL affinity. The substitution of the chlorine in the structure of **G1** with benzyl or phenylsulfide, but not phenylsulfone, improved the compounds’ potency. The addition of two fluorine atoms to the N-benzoyl fragment led to compound **G3** (Figure 8), a potent and reversible MAGL inhibitor with low effects on FAAH and ABHD6 [52]. Compounds **G4** and **G5** were obtained after testing various substituents on the phenylsulfide fragment of **G3** [16].

Tabrizi et al. identified the 1,5-diphenylpyrazole-3-carboxamide scaffold as an option for further development. Several rounds of structural investigations were carried out by maintaining a fixed central pyrazole core and varying the substituent present on the nitrogen atom (phenyl or methyl group) and the fragment linked to the carbonyl group in position 3. Derivatives with a *p*-hydroxyl group on the benzene attached in position 5 possessed higher inhibitory activity compared with other substitution patterns. The hydroxyl permits the formation of an H-bond with the oxygen of Pro178. The best result was achieved by linking the carbonyl with the nitrogen of 4-benzylpiperidine, as can be seen in the structure of compound **G6** (Figure 9). The elimination of the *p*-hydroxyl or its replacement with a chlorine atom reduces the potency, demonstrating its role in the inhibition of MAGL. The next optimization stage focused on changing the 1-pyrazole substitution with different aromatic moieties, such as the benzyl, phenylethyl or phenylpropyl. Compound **G7** emerged as the most active of this series of derivatives. The docking studies indicated that *m*-methylbenzyl connected to pyrazole is oriented to the surface of the binding site and forms lipophilic interactions with Ala151 and Leu241. The compound can be considered to be a structural analogue of **G1** [48].

The compound **G7** is relatively selective towards other endocannabinoids, degrading enzymes producing a 7% inhibition of FAAH at 10 μM and presenting no significant binding affinities the cannabinoid receptors. 

Zhi et al. (2020) designed amide derivatives of 1-benzoylpiperidine as selective and reversible MAGL inhibitors. Compound **G8** (Figure 9) emerged as a first lead structure. The docking studies indicated the importance of the carbonyl in position 1 of the piperidine ring by forming two H-bonds with the nitrogen backbone of Ala51 and Met123. The chlorine atom showed hydrophobic interaction with Val191 and Tyr194, and its presence is correlated with an increased inhibitory potency. When the chorine was replaced with a hydroxyl group in the structure of **G8**, the inhibitory activity was reduced significantly.

The optimization process led to a further increase in the inhibitory effect, particularly when a larger hydrophobic group was attached to the carbonyl (naphtalen vs. benzene), allowing a better occupancy of the larger hydrophobic cavity and the establishment of various lipophilic interactions. The *m*-chloroaniline fragment of **G9** bonds to the hydrophobic subpocket enclosed by side chains of Val191, Tyr194, Val270 and Lys273, whereas the naphthalene moiety forms lipophilic interactions with Leu148, Leu213 and Leu241 [49]. **G9** was selective towards MAGL and presented no significant binding affinities with the cannabinoid receptors. It demonstrated a very low toxicity in the mouse fibroblasts (L929) assay.

#### 4.2.3. Pyrrolidone Derivatives

A high-throughput screening identified 1-(4-phenoxyphenyl)-2-pyrrolidone as a weak inhibitor of MAGL. Adding a pyrimidinyl piperazine fragment to the 2-pyrrolidone ring led to compound **H1** having a significantly increased inhibitory effect. The pyrimidinyl piperazine scaffold was previously used in other types of MAGL inhibitors, such as the compound **A3** (Figure 3). Using a structure-based drug design approach, a novel series of reversible MAGL inhibitors was developed, starting from the lead piperazinyl-pyrrolidin-2-one core [27].

**Figure 10:**
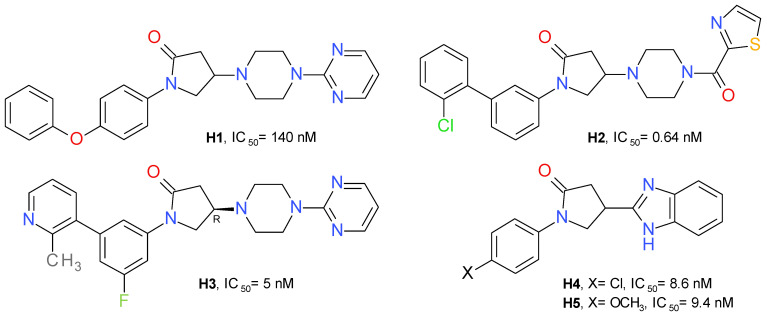
Reversible MAGL inhibitors—pyrrolidone derivatives.

Changing the pyrimidine ring with benzene or benzoyl proved to be unfavorable. The 2-thiazole-carbonyl fragment provided an approximately 3.5-fold increase in potency. This fragment was selected to occupy the amphiphilic pocket of the enzyme and used in further investigations of this series. The next step was focused on the nature of the N-pyrrolidine substituent. The conformational change of 4-phenoxyphenyl to its 3-phenoxyphenyl isomer improved the inhibitory effect on MAGL. Among the derivatives with substituents at position 3 of the central benzene ring, the chloro-derivatives showed the best profile. Despite the high potency of compound **H2** (Figure 10), it showed a low metabolic stability. To improve the metabolic stability, a series of successive structural modulations led to the optimal biaryl moiety, the 3-fluoro-5-(2-methylpyridin-3-yl)phenyl fragment. The authors returned to the initial pyrimidinyl piperazine scaffold and obtained the compound **H3** [27].

Oral administration of **H3** (10 mg/kg) lead to a 25% decrease in AA concentration and a 340% increase in 2-AG in the brain of mice, compared to the control [27].

A series of variously substituted N-phenyl-pyrrolidin-2-one linked with benzothiazol or benzimidazole was synthesized and assayed on human MAGL. The benzimidazole derivatives produced better inhibition on MAGL. Compound **H4** determined a potent inhibition on MAGL with an IC_50_ value of 8.6 nM, and its methoxy analogue, compound **H5** (Figure 10), presented a similar inhibitory profile, but a better selectivity towards FAAH [53]. Structurally similar compounds were prepared linking benzoxazole in the position 4 of the pyrrolidone scaffold. The best inhibitory effects were observed for the compounds substituted at the nitrogen atom with 4-nitrobezene and 4-benzenesulfonamide [54].

#### 4.2.4. Azetidinyl Amides

**Figure 11:**
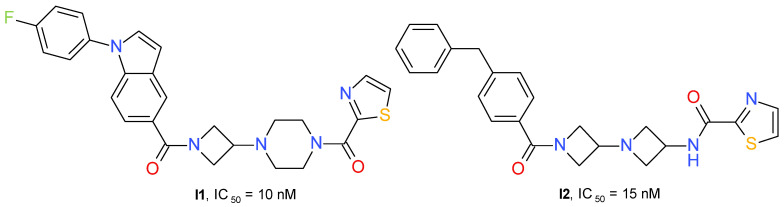
Reversible MAGL inhibitors—azetidinyl amide derivatives.

Zhu et al. (2020) developed a series of 3-piperazinyl-azetidine diamides with potent inhibitory effects on MAGL. The derivatives containing a 2-thiazole-carbonyl fragment connected to piperazine were, in general, more active than the compounds having a benzoyl fragment. The lipophilicity of the azetidinyl amide substituent influenced the MAGL inhibitory potential. The best compound was chosen based on the IC_50_ value and the percentage 2-AG brain accumulation measured in homogenized rat brain incubated with 1 μM of each test substance. Compound **I1** (Figure 11) has little effect on FAAH (IC_50_ = 4 μM) and no significant interaction with receptors CB1R or CB2R. 

A structurally similar series was developed using the diazetidinyl diamide scaffold. The compounds can be considered to be 3-aminoazetidine equivalents of the piperazine derivatives. The thiazole derivatives group presented higher potency in the MAGL enzyme assay than the corresponding analogs containing a phenyl fragment. The thiazole-amide of **I2** (Figure 11) forms a hydrogen bond to the side chain guanidine group of Arg57 and a π-π stacking interaction with Tyr194. The existence of an azetidine-amide carbonyl allows access into the oxyanion hole of MAGL and forms a hydrogen bond with the backbone amide NH of Met123, adjacent to the catalytic Ser122 [55].

#### 4.2.5. Various Structures

**Figure 12:**
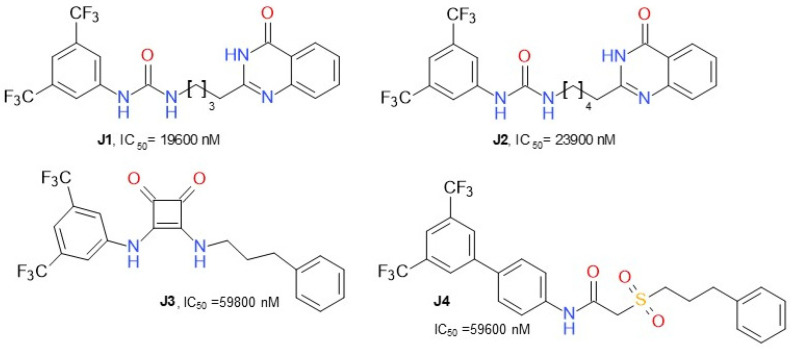
Reversible MAGL inhibitors—various structures.

Dato et al. (2020) developed various compounds (Figure 12) targeting MAGL and presented ω-quinazolinonylalkyl aryl ureas as a new class of inhibitors of MAGL, exhibiting IC_50_ values in the range of 20–41 µM. The compound **J1**, 1-(3,5-bis(trifluoromethyl)phenyl)-3-(4-(4-oxo-3,4-dihydroquinazolin-2-yl)butyl)urea, was the most active inhibitor. This class of compounds was shown to interact with MAGL in a reversible manner, exhibiting a mixed-type inhibition with a predominant competitive behavior [56]. 2-sulfonacetamide (**J4**) and squareamide (**J3**) derivatives possessed lower IC_50_.

## 5. Potential Therapeutic Applications of MAGL Inhibition

As presented above, MAGL cleaves 2-AG and other monoacylglycerols [12,16], thus influencing the levels of several other lipid molecules with pro-inflammatory and pro-neoplastic functions. In addition, 2-AG activates several other receptors, leading to intricate, interconnected signaling pathways [12]. Consequently, MAGL is emerging as a multifaceted therapeutic target for a steadily increasing list of maladies, some of which are currently lacking targeted or highly efficient curative treatments [12,16].

MAGL inhibition is associated with neuroprotective effects [12,16], partly by reducing central pro-inflammatory mediators’ levels (discussed below), and partly by enhancing 2-AG activation of CB1R/ CB2R receptors and of down-stream signaling pathways [57].

Because it catalyzes the formation of AA from 2-AG, MAGL can be included in the category of pro-inflammatory enzymes. As a major cerebral source of AA, it is linked to the synthesis of prostaglandin E2 (PGE_2_) in both basal and pro-inflammatory settings. Mice lacking MAGL exhibited highly elevated brain 2-AG levels with concomitant decreases in arachidonic acid levels, and its oxidative metabolites PGE_2_ and PGD_2_, indicating MAGL is a key player in neuroinflammatory responses [10,58]. Chronic inflammation is a central process involved in a high number of metabolic disorders, neurodegenerative and neurological, malignant diseases, and autoimmune diseases [59].

CB1R activation modulates neurotransmitter release, neurogenesis, and synaptic plasticity, and affects neuronal functions through interaction with glial cells. 2-AG can activate astrocytic CB1R receptors, leading to neurotransmitter release, thus modulating neuronal functions beyond the originating synapse [60]. In addition, CB1R receptor activation was shown to activate the phosphatidylinositol 3-kinase (PI3K)/protein kinase B (Akt)/mammalian target of the rapamycin (mTOR) complex 1 pathway, which, in turn, induces brain-derived neurotrophic factor (BDNF) expression [57]. Moreover, the activation of CB1R receptors (by 2-AG) can induce autophagy, facilitating the elimination of protein aggregates [61].

CB2R activation decreases microglial activation and neuroinflammatory response [62]. Furthermore, 2-AG was shown to modulate other substrates such as peroxisome proliferator-activated receptors (PPARs, NR1C3) [63,64] or the G-protein receptor 55 (GPR55) [65], receptors found to reduce glial reactivity, and consequently local inflammatory events.

Genetic animal models showed MAGL deletion inhibits the progression of neurodegenerative diseases such as Huntington disease [66], Parkinson disease [10] and Alzheimer disease [67].

The elements of the cannabinoid system are expressed almost ubiquitously throughout nociceptive pathways, indicating the system modulates nociceptive signaling [68]. Genetic deletion of MAGL significantly increases central and peripheral 2-AG, leading to the downregulation and the desensitization of central and peripherally expressed CB1R receptors. CB1R desensitization was observed in brain areas controlling emotional and stress-related states, pain sensation, memory and learning, whereas brain areas associated with motor function were not affected by it. Consequently, genetic mouse models of MGL deficiency do not show analgesic properties [69,70]. By comparison, partial pharmacological MAGL inactivation resulted in significant analgesic effect in various models of pain, as further discussed below.

Modulation of endocannabinoids’ levels may offer an alternative for the treatment of psychiatric disorders, as a consequence of CB1R-mediated reduction of neuroinflammation and of the dopaminergic system modulation [71]. Neuronal and glial-derived endocannabinoid-signals are associated with cognitive dysfunction and with the pathogenesis of epilepsy [58], making MAGL a pharmacological target for epilepsy treatment.

MAGL is involved in energy balance by mobilizing cellular lipid stores in adipose and other tissues [72] and by regulating 2-AG concentration [17]. CB1R activation by 2-AG determines energy accumulation [17]. Centrally, it potentiates hypothalamic orexigenic pathways, and peripherally it enhances fat uptake in adipose tissue, increases de novo lipogenesis in the liver and decreases energy expenditure in muscle [17].

In diet-induced obesity models, MAGL ablation prevented the development of glucose intolerance and insulin resistance [73]. No changes in the body weight of MAGL−/− mice after 12 weeks of very high-fat feeding were observed, suggesting the desensitizing effects of cannabinoid receptors [73]. The results were further confirmed by other authors, who also underlined the liver content of certain species of saturated and unsaturated MAGs were highly enriched in MAGL−/− mice [72,74]. In addition to producing a leaner phenotype, global MAGL deletion led to an improved serum metabolic profile [72].

Switching to a lipogenic phenotype is part of the multitude of metabolic changes accompanying a cell’s malignant transformation [75]. The increased expression of MAGL in aggressive malignant cells was linked to its ability to modulate a network of pro-oncogenic lipids [16,75,76,77]. MAGL overexpression by non-aggressive cancer cells, which can be achieved following a high-fat diet, alters their phenotype, increasing malignancy. These effects can be reversed in vitro, by administering a MAGL inhibitor [48,50,78]. MAGL inhibition was shown to decrease proliferation and increase apoptosis, via the upregulation of Bax and downregulation of Bcl-2 and Cyclin D1 expression [47,49].

MAGL was found to be upregulated in androgen-independent prostate cancer cells [79], malignant melanoma cells [76] and hepatic carcinoma cells [80,81], with pro-tumorigenic effects, by modulating both endocannabinoid and fatty acid metabolism/signaling. Its inhibition led to a decrease in cancer aggressiveness, which was reversed by a CB1R receptor antagonist or fatty acids administration [79].

### 5.1. Irreversible Pharmacological Inhibition of MAGL

Irreversible MAGL pharmacological inhibition in rodents was shown to have a great utility in addressing various disorders, supporting the hypothesis that MAGL represents an attractive therapeutic target.

#### 5.1.1. Diseases of the Central Nervous System

KML29 (Figure 2) delayed the onset and progression of amyotrophic lateral sclerosis by reducing inflammation and increasing BDNF expression [82]. MAGL inhibition (JZL184-Figure 2) in primary mouse striatal astrocytes resulted in a decreased mutant huntingtin-induced synthesis of TNF-α via CB1R receptor activation [66]. Further, blocking MAGL expression led to cells resistant to mutant huntingtin-induced neuronal loss [66].

MAGL inhibition resulted in the suppression of β-amyloid synthesis and accumulation, and decreased β-site amyloid precursor protein cleaving enzyme 1 (BACE1), in a mouse model of Alzheimer’s disease. Due to reducing neuroinflammation and neurodegeneration, the hippocampal synaptic structure and function was maintained, and long-term synaptic plasticity, spatial learning and memory were improved [83,84,85].

Thus, MAGL inhibition promotes neuron survival in chronic neurodegenerative disorders, a property that preclinical studies show can also be extended to acute degeneration conditions (e.g., stroke and brain trauma). In a middle cerebral artery occlusion model of stroke, the administration of the MAGL inhibitor JZL184 (Figure 2), alone or in combination with the tissue plasminogen activator, determined the reduction of brain edema and infarct volume, reducing neuronal loss, in a CB1R-dependent manner [86]. The same inhibitor prevented neuropathologic changes and promoted neurologic recovery in a closed head injury model [62].

The blockade of MAGL resulted in anxiolytic effects in mice [87,88] and rats [89]. Regarding stress-induced anxiety, MAGL inhibition resulted in improved excitatory-inhibitory balance in the ventromedial prefrontal cortex, promoting traumatic stress resilience in rats [90]. Stress-related mental illness was associated with neuroinflammation, and stress-induced behavior was linked to PGE2 synthesis. In a social defeat stress model, PGE2 synthesis in the subcortical region, but not the cortical region, was linked to the activities of MAGL and COX-1/2 in a TLDR2/2-dependent manner [91].

Both anxiolytic and antidepressant effects observed are partly secondary to increased corticosterone secretion, whereas the enhanced locomotor activity is an intrinsic effect of augmented 2-AG signaling [92]. MAGL inhibitors were reported to exert a significant antidepressant effect, as reported in chronic corticosterone-exposed mice via GABAergic synaptic disinhibition [92].

MAGL inhibitors reduce symptoms associated with chronic stress such as hyperalgesia [88] and reduce intestinal permeability, the latter via modulation of claudins 1, 2 and 5, and occludin expression [93].

MAGL inhibition was useful in reducing seizures in both status epilepticus [94] and focal seizures [95] models. It prevented associated neuronal cell loss and cognitive impairment, in addition to reducing seizure-induced IL-1β and COX-2 expression [94].

Inhibition of MAGL is a viable target in treating nicotine addiction. A significant correlation between enzyme expression and withdrawal intensity was found in a murine model. Furthermore, administration of the JZL184 inhibitor (Figure 2) dose-dependently reduced withdrawal signs in a CB1R-dependent manner [96].

KML29 (Figure 2), administered systemically, produces six-fold elevations in brain 2-AG without markedly elevating AEA levels, and possesses anti-allodynic effects in a chronic constriction injury model [97].

Significant antinociception has been demonstrated in rodent models of peripheral inflammatory pain [55,95,98,99], visceral and gastrointestinal pain [95,100], neuropathic pain [97,101,102] and chemotherapy-induced neuropathy [103,104]. The selective inhibitor B3 (Figure 4) proved useful in suppressing pain-associated behavior in a rat formalin pain model [35]. KML29 (Figure 2) resulted in significantly lower inflammation and pain in a monoiodoacetate model of osteoarthritis, with reduced leukocyte infiltration, joint pain, withdrawal threshold and development of secondary allodynia [98]. In a similar osteoarthritis model, treatment with the inhibitor MJN110 (Figure 2) reversed weight-bearing asymmetry, lowered paw withdrawal thresholds (acute treatment) and resulted in anti-nociceptive tolerance (repeated treatment) [105]. MJN110 also blocked the expression of membrane-associated PGE synthase-1 in the ipsilateral dorsal horn of the spinal cord of the monoiodoacetate-treated rats [105]. Various inhibitors reduced chronic constriction, injury-induced and paclitaxel-induced mechanical allodynia, and thermal hyperalgesia, as a consequence of CB1R/CB2R activation [89,90,91]. In models of acute pain, effects of MAGL inhibition appear to be largely mediated by CB1R, whereas in inflammatory and neuropathic pain models, the analgesic effect is based on the activation of both CB1R and CB2R.

#### 5.1.2. Inflammatory Diseases

Pharmacological inhibition of MAGL leads to reduction of neuroinflammatory responses in a manner associated with 2-AG increase and with AA, IL1β, IL6 or TNFα decrease in various animal models, such as LPS-induced neuroinflammation [106], MPTP-induced Parkinson, and genetic models of Alzheimer and SLA [62,82,106]

Nonetheless, the brain region-specific effects of MAGL should be considered when targeting its pharmacologic inhibition, because pro-inflammatory outcomes were reported. In mice with genetic deletion of the enzyme or treated with a specific inhibitor (JZL184), microglial reactivity was observed in the cerebellum, but not in the hippocampus. This resulted in increased mRNA for pro-inflammatory molecules, such as the enzyme COX-2, and impairment of motor coordination [107].

Inhibition of MAGL generation of AA exerted an CB1R receptors-independent antipyretic effect in mice, in an IL-1β- or LPS-induced fever model [108]. However, MAGL deletion is not required for the maintenance of the basal CBT and temperature homeostasis, but peripherally attenuates induced fever response [108].

A milder but still significant increase in 2-AG levels and in other monoacylglycerols was observed in the thymus, spleen and liver of MAGL KO mice [58], supporting the importance of MAGL in the regulation of the peripheral inflammatory response.

MAGL inhibitors exerted beneficial effects in animal models of chronic inflammatory illnesses, such as complete Freund reactive induced arthritis [98] and collagen-induced arthritis [99].

In mice fed a Western diet, MAGL knockout led to decreased hepatic inflammation [109]. MAGL deletion had protective effects on hepatocytes in different models of acute liver injury, an effect mediated via 2-AG CB2R-enhanced signaling and modulation of eicosanoid pathways, leading to a decrease in neutrophil-mediated liver damage [109]. Similar outcomes were observed in a preclinical model of hepatic injury associated with inflammation [110]. Furthermore, enhancement of CB2R activation appears to prompt fibrosis regression due to autophagy-mediated anti-inflammatory mechanisms in macrophages [109].

Regarding skeletal muscle post-injury healing, the MAGL inhibitor JZL184 (Figure 2) yielded an anti-inflammatory effect, decreasing neutrophil and macrophage infiltration and pro-inflammatory cytokine expression. However, this resulted in a delayed myofiber regeneration and promotion of fibrosis. These effects were reported to be CB1R receptors- and CB2R receptors-dependent [111].

The use of the MAGL inhibitor URB602 (Figure 2) was tested in a mice model of lung ischemia-reperfusion injury, which is a postoperative complication that can occur post-lung transplantation/cardiopulmonary bypass. Pre-treatment resulted in several protective effects: decreased wet to dry lung weight ratio score and improved oxygenation index, in addition to increased levels of 2-AG and decreased levels of PGI2, TXB2, LTB4, IL-6 and TNF-α [112]. In an LPS-induced acute lung injury murine model, MAGL inhibition with JZL184 decreased leukocyte migration, vascular permeability, and cytokine/chemokine and adhesion molecules levels in the bronchoalveolar lavage fluid [113].

#### 5.1.3. Other Possible Applications

##### Obesity and Metabolic Diseases

Preclinical studies with MJN110 (Figure 2) showed that MAGL inhibitors’ administration is associated with reduced food intake in rats [114]. Taking into account the above-mentioned effects, and also that systemic oxidative stress, which is reported to possess a negative impact on lipids, proteins and endothelial function [115], is decreased by 2-AG [116], inhibiting MAGL may prove a useful strategy for the treatment of obesity and its comorbidities.

##### Neoplastic Maladies

Preclinical studies have confirmed the results of the in vitro studies, with JZL184 (Figure 2) showing antiproliferative efficacy in breast and prostate cancers, and in osteosarcoma [117], consequent to CB1R/CB2R activation and a decrease in inflammatory markers. Furthermore, in a xenograft and azoxymethane-induced colon cancer model, MAGL inhibition led to downregulation of VEGF and FGF-2, thus justifying the antiangiogenic and antimetastatic effect determined by MAGL inhibition.

All preclinical studies presenting the therapeutic utility of the irreversible MAGL inhibition are summarized in Table 1.

One aspect to be taken into account relates to the inhibitors’ selectivity towards MAGL rather than other endocannabinoid processing enzymes such as FAAH or ABHD6. MAGL inhibitor JZL184 (Figure 2) has been shown to have low specificity and thus blocking activities of other hydrolases such as fatty acid amide hydrolase and carboxylesterases [58], producing an increase in the central levels of 2-AG and OEA [87]. In consequence, repetitive, high doses of this inhibitor, e.g., 40 mg/kg [103,104], due to the persistent cerebral endocannabinoids, increase impaired endocannabinoid-dependent synaptic plasticity, leading to physical dependence and desensitization of brain CB1R receptors. The partial blockade of MAGL using low doses (4 mg/kg) of JZL184 for ~7 days did not lead to CB1R receptor desensitization or behavioral tolerance [103]. It was reported to decrease locomotor activity but did not induce catalepsy and hypothermia [101].

Compounds with higher MAGL selectivity generally lack cannabinoid adverse effects. Thus, KML29, an inhibitor with higher selectivity than JZL184, did show analgesic tolerance at high doses but did not induce locomotor activity decrease, tail immersion, or body temperature differences vs. control [97], nor catalepsy or hypothermia [101].

The tolerance observed in preclinical trials may explain the results of the clinical trials assessing the efficacy of **B3** (Figure 4; currently LuAg06466, known formerly as ABX-1431), a highly potent and selective MAGL inhibitor, suitable for once-per-day oral administration, in Tourette syndrome [41]. When tested in a randomized, double-blind, placebo-controlled crossover, exploratory phase 1b study, a single dose was shown to improve tics and premonitory urges in adults with this disease [126]. However, administration of this compound for a longer period of time (12 weeks) showed no improvement in tic severity, premonitory urges, quality of life, and common psychiatric comorbidities when compared with a placebo [126].

It is hypothesized that using reversible inhibitors will improve the safety profile, preventing the manifestation of the adverse reactions mentioned above, including that of tolerance [1].

### 5.2. Reversible Pharmacological Inhibition of MAGL

Although various reversible inhibitors were identified, information from preclinical testing is scarce and further testing is needed, as seen in Table 2. The reversible inhibitors show pharmacodynamic effects similar to those of irreversible inhibitors in neuropathic and inflammatory pain [47,53,55] and for ameliorating characteristics of reserpine-induced depression [49]. In a previous study using a multiple sclerosis murine model, a reversible and selective MAGL inhibitor proved useful in stalling disease progression, with no CB1R receptors-related undesired effects [47]. We must underline that, for G7, repeated treatment was also tested: this reversible MAGL inhibitor showed complete lack of tolerance development, with long-lasting efficacy of the compound after repeated treatments.

**Figure 13:**
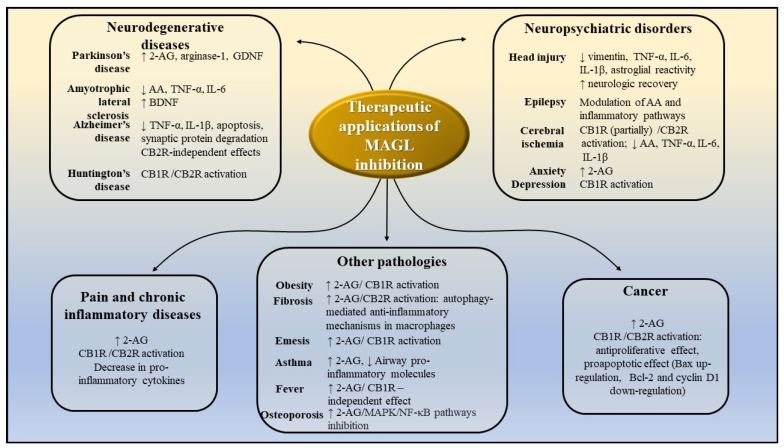
Potential therapeutic application of MAGL inhibitors, as demonstrated by preclinical studies. AA-arachidonic acid; 2-AG-2-arachidonoylglycerol; CB1R—cannabinoid receptors 1; CB2R—cannabinoid receptors 2; IL-1β-interleukin-1β; IL-6—interleukin 6; TNF-α -tumor necrosis factor α; MAPK- mitogen-activated protein kinase; NF-kB—nuclear factor kappa-light-chain-enhancer of activated B cells; GDNF—glial cell line-derived neurotrophic factor; BDNF—brain-derived neurotrophic factor.

Figure 13 summarizes the potential therapeutic applications of MAGL inhibitors and portrays the potential mechanisms of action, based on the results of preclinical studies presented above.

## 6. Conclusions

Several preclinical studies showed beneficial effects of MAGL blockades in various disorders, such as neurodegenerative and refractory neuropsychiatric disorders, chronic pain, and cancer. Thus, synthesizing selective MAGL inhibitors has become a focus point in drug design and development.

In the present review, we summarized the diverse synthetic scaffolds of MAGL inhibitors reported between 2015 and 2021, in connection with their potency. We presented their structural features critical for high selectivity and increased inhibitory potency, with the purpose of offering new insight for the development of novel inhibitors.

Irreversible inhibitors—developed from scaffolds such as hexafluoroisopropyl alcohol carbamates, glycol carbamates, azetidone triazole ureas and benzisothiazolinone—possess sustained pharmacological response and increased potency, whereas reversible inhibitors—derivatives of salicylketoxime, piperidine, pyrrolidone and azetidinyl amides—are expected to ensure a superior safety profile.

By modulating both endocannabinoid signaling and arachidonic acid metabolism, MAGL represents an attractive target in medicinal chemistry, and its inhibition may prove key to treating various pathologies currently lacking a satisfactory treatment. However, one should note that, due to the complexity of the biological networks in which MAGL functions, the extent of the consequences and effects generated by its pharmacological inhibition cannot be fully understood.

## Figures and Tables

**Table 1:** Preclinical studies assessing MAGL irreversible inhibitors’ utility, conducted between 2015 and 2021.

Therapeutical Application	Compound	Animal Model	Animal Species	Results	Alleged Mechanism	Reference
Pain and inflammatory disorders						
Neuropathic pain	JZL184	Neuropathic pain induced by trigeminal nerve injury	C57BL/6J mice	Attenuates orofacial neuropathic pain.	Not mentioned	[97]
	MJN110 JZL184	Chronic constriction injury model	C57BL/6J mice	Reduced chronic constriction injury-induced mechanical allodynia and thermal hyperalgesia.	CB1R/CB2R activation Decreased whole-brain arachidonic acid levels, no change in AEA, OEA, or PEA levels	[101]
	JZL184	Neuropathic pain induced by trigeminal nerve injury	C57Bl/6J mice	Attenuates orofacial neuropathic pain.	Not mentioned	[102]
	MJN110	Paclitaxel-induced neuropathic pain	C57BL/6J mice	Reverse paclitaxel-induced mechanical allodynia. Prevents increased expression of MCP-1 and p-p38 MAPK in dorsal root ganglia as well as MCP-1 in spinal dorsal horn.	CB1R/CB2R activation	[103]
	JZL184	Paclitaxel-induced neuropathic pain	C57BL/6J/ BALB/c mice	Reverse paclitaxel-induced mechanical allodynia.	CB1R/CB2R activation	[104]
Inflammatory pain/other inflammatory disorders	KML29	Monoiodoacetate-induced osteoarthritis	Wistar rats	Analgesic effect—reduces secondary allodynia. Anti-inflammatory effect characterized by a decrease in rolling and adherent leukocytes.	CB1R/CB2R activation	[98]
	SAR127303	Formalin-induced and phenylbenzoquinone -induced pain	CB1R7 SCID mice	Antinociceptive effects Alters learning performance in several assays related to episodic, working and spatial memory.	CB1R (visceral pain). None reported for inflammatory pain Elevates hippocampal levels of 2-AG in mice, not AEA, PEA and OEA levels	[95]
	MJN 110	Monoiodoacetate-induced osteoarthritis	Sprague Dawley rats	Analgesic effect—reduces secondary allodynia. Anti-inflammatory effect.	CB1R/CB2R activation	[105]
	JZL184	Colonic distension model/acetic acid-induced pain	BALB/c mice	Alleviate pain-related behaviors.	Not mentioned	[100]
	JZL184	Collagen-induced arthritis	DB1A mice	Reduced paw inflammation and pain-depressed behavioral signs. Dose-dependently attenuated grip strength and balance beam deficits caused by arthritis.	CB2R activation	[99]
	B3 (Figure 4)	Formalin-induced pain	Sprague-Dawley rats	Dose-dependent reduction of pain response in both acute and late phases. No reduction in motor activity.	2-AG increase	[34]
	JZL184	Collagen-induced arthritis	DB1A mice	Reduced paw inflammation and pain-depressed behavioral signs. Dose-dependently attenuated grip strength and balance beam deficits caused by arthritis.	CB2R activation	[99]
	B3 (Figure 4)	Formalin-induced pain	Sprague-Dawley rats	Dose-dependent reduction of pain response in both acute and late phases. No reduction in motor activity.	2-AG increase	[34]
Neurodegenerative diseases						
Parkinson disease	KML29	MPTP/probenecid-induced Parkinson	C57BL/6J mice	Attenuated striatal dopamine depletion.	Striatal 2-AG, arginase-1 and GDNF increase	[118]
Amyotrophic lateral sclerosis	KML29	Genetic model	Low-copy SOD1G93A mice	Slows down onset, progression, increases survival. Delays the decrease in body weight and in motor activity Neurotrophic and anti-inflammatory effects.	AA, TNFα and IL6 decrease BDNF increase in spinal cord Effects are not PG-related	[82]
Alzheimer’s disease	JZL184	Genetic model	APdE9 mice	Marked decrease in total Aβ burden in the temporal and parietal cortex and, to some extent, in the hippocampus. Decreased the pro-inflammatory reactions of microglia.	Not mentioned	[83]
	JZL184	Genetic model	5XFAD APP/5XFAD APP-CB2R-KO transgenic mice	Reduces neuroinflammation and neurodegeneration. Improvements in spatial learning and memory decrease in the expression of APP and β-secretase as well as production of total Aβ and Aβ42.	Not mediated via CB2R. Other receptors. Prevents deterioration in expression of synaptic proteins (PSD95, AMPA receptor subunits GluA1 and GluA2, and NMDA receptor subunit GluN2B).	[84]
	JZL184	Genetic model	tau P301S/PS19 transgenic (TG) mice	Suppressed inflammatory responses in astrocytes and reactive microglial cells in the cortex and hippocampus. Decreased tau neuronal loss.	Decreased hippocampal IL-1β and TNFα. Prevented the increase in p-GSK3β, P35/25, p-NF-kB. expression and the decrease in expression of PPARγ. Inhibited apoptosis through a caspase-3-dependent signaling pathway.	[85]
Huntington disease	JZL184	Knock-in mouse model	Q175 mice	Prevents motivational deficit. Increases dopamine release during a progressive-ratio task.	CB1R/CB2R activation	[119]
Autoimmune encephalomyelitis/demyelination	JZL184 D1 (Figure 6) C5 (Figure 5)	Immunization with myelin oligodendrocyte glycoprotein in incomplete Freund’s adjuvant, lipopolysaccharide (LPS) or cuprizone-induced demyelination.	C57BL/6J mice	Protects oligodendrocytes from excitotoxicity, thus protecting white matter. Attenuates neurological deficits and/or prevents myelin loss.	2-AG increase. CB1R activation. Other receptors Decrease in AMPA-induced cytosolic calcium overload, mitochondrial membrane depolarization, and production of reactive oxygen species. Prevented LPS-induced increase in TNFα, PGE_2,_ IL-1β.	[26,42,120]
Neuropsychiatric disorders						
Head injury	JZL184	Repetitively Mild Closed Head Injury model	C57BL/6 mice	Reduces chronic traumatic encephalopathy-like neuropathologic changes (impairments in basal synaptic transmission, long-term synaptic plasticity, and spatial learning) and promotes neurologic recovery. Decreases expression of APP and the enzymes that synthesize Aβ, production of Aβ, neurodegeneration, aggregation of TDP-43 protein and phosphorylation of tau.	Pro-inflammatory markers vimentin, IL-1β, IL-6, and TNFα) decrease and reactivation of astroglial cells inhibition	[121]
Focal cerebral ischemia model	JZL184	Endothelin-1-induced, transient or non-transient occlusion of the middle cerebral artery ischemia	Wistar-Kyoto rats	Attenuated infarct volume and hemispheric swelling, sensorimotor deficits, inflammatory response, and decreased the number of degenerating neurons. Decrease in microglial activation and neuroinflammatory response.	CB2R activation/Partially CB1R activation (sensory impairment) Other receptors Significant TNF-α microglial reduction	[62]
Stroke	JZL184	Permanent cerebralischemia model	Mice Strain not specified	Lowered brain infarction, reduced brain edema, improvement of behavioral functions.	CB1R activation 2-AG, IL-10 increase AA, MMP9, TNF-α decrease	[86]
Inflammatory and ischemic blood-brain barrier disruption	CPD-4645	LPS-induced and ischemic-induced blood-brain barrier disruption	C57BL/6 mice	Reduced blood-brain barrier damage in both models. Prevented neuroinflammation.	CB1R/CB2R activation, in ischemic model Other receptors 2-AG increase AA, IL1β and IL6 decrease	[106]
Epilepsy—status epilepticus (SE)	CPD-4645	Diazepam-resistant SE model	C57BL6N mice/(Cnr1−/−) mice	Reduces benzodiazepine-refractory SE and prevents cell loss and cognitive deficits.	Independent on CB1R receptors Modulation of arachidonic acid and inflammatory pathways	[94]
Epilepsy—focal seizures	SAR127303	Corneal kindling-induced seizures	CB1R7 SCID mice	Inhibits seizure initiation and protects against focal seizure activity.	Elevates hippocampal levels of 2-AG in mice, not AEA, PEA and OEA levels	[95]
Anxiety/depression	KML29	Chronic corticosterone-induced stress	CD1 or C57BL/6 mice	Antidepressant effects on acute stress-exposed mice, through astrocyte-mediated glutamatergic synaptic long-term depression (low dose), rapid and long-lasting antidepressant responses in chronically stressed mice likely through disinhibition of GABAergic synapses (high dose).	2-AG increase, consequent CB1R activation	[92]
	JZL184	Early life stress model	Sprague Dawley pups	Prevented depression- and anxiety-like behavior and the impairment in social behavior and neuronal Plasticity. Prevented induced alterations in BDNF hippocampus and in nucleus accumbens.	Partially via CB1R activation	[89]
	JZL184	Non-stress and stress (foot-shock, restraint)-induced anxiety	ICR (CD-1) mice	Prevents anxiety-like behavior in rodents.	Not mentioned.	[87]
	JZL184	Chronic unpredictable stress-model	C57BL/6J mice	Reduce chronic unpredictable stress-induced anxiety and thermal hyperalgesia.	Increase in 2-AG	[88]
	KML29	Stress-induced anxiety	Fischer-344 rats	MAGL inhibition in the ventromedial prefrontal cortex augments the output of neurons that project to brainstem and limbic structures that mediate stress responses, preventing stress-induced anxiety.	Increase in 2-AG	[90]
Cancer						
Breast and prostate cancers Osteosarcoma	JZL184	Genetic model	C57BL/6 J and BALB/c-nu/nu mice	Decrease in cancer-related bone damage (osteoprotective effect), reduced skeletal tumor growth and metastasis, suppressed cachexia, prolonged survival.	2-AG increase consequent CB1R/CB2R activation and inflammatory markers decrease	[117]
Colon cancer		Xenograft and azoxymethane-induced colon cancer	ICR mice	Attenuated azoxymethane-induced preneoplastic lesions, polyps and tumors and reduced xenograft tumor volume. Antiangiogenic effect.	Down-regulation of VEGF and FGF-2, reduction in the number of vessels and down-regulation of cyclin D1	[122]
Other pathologies						
Fibrosis	MJN110	Carbon tetrachloride-induced liver fibrosis	C57BL/6J/(MAGL−/−) mice	Reduced hepatic macrophage number, inflammatory gene expression and slowed down fibrosis progression. Accelerated fibrosis regression.	Reduces the production of IL-1α, IL-1β, PGE2 and TXA2 from macrophages, via an autophagy-dependent pathway (independently of CB2R receptors)	[110]
Emesis	MJN110	Taste reactivity model—LiCl-induced acute vomiting and contextually elicited anticipatory gaping	Sprague– Dawley rats	Suppressed both acute and anticipatory nausea.	2-AG increase consequent CB1R activation	[123]
Emesis	AM4301	Taste reactivity model	Sprague-Dawley rats	Suppressed acute nausea, when delivered systemically or into the interoceptive insular cortex.	CB1R-mediated	[124]
Asthma	JZL184	LPS-induced airway inflammation	CD1 mice	Prevents increased serotonin-induced contractions and reduces peribronchial and parenchymal inflammation	2-AG increase Reduces airway TNF-α, IL-1β	[113]
Lung ischemia	URB602	Lung ischemia-reperfusion injury model	C57BL/6 mice	Preventive or therapeutic regimen reduced lung injury score while increased oxygenation.	2-AG increase Reduces airway pro-inflammatory mediators AA, PGI2, TXB2, LTB4 and inflammatory citokines IL-6, TNF-α	[112]
Fever	JZL184	Centrally and peripherally-administered LPS or IL-1β-induced fever models	*MAGL* −/− and *MAGL* +/+ mice	Reduces fever (but it does not suppress it). Does not alter normal temperature.	2-AG increase Not mediated via CB1R	[108]
Osteoporosis	JZL184	Ovariectomized mouse model	*C57BL*/6 *mice*	Ameliorated bone loss. Suppressed osteoclast differentiation, bone resorption, and osteoclast-specific gene expression.	MAPK and NF-κB inhibition	[125]
Muscle contusion	JZL184	Sprague Dawley rats	Rat muscle contusion model	Decreases inflammatory response in skeletal muscle contusion in rats: decreased neutrophil and macrophage infiltration and pro-inflammatory cytokine expression.	CB1R/CB2R activation 2-AG increase Decreased arachidonic acid levels and pro-inflammatory cytokines	[111]

2-AG—2-arachidonoylglycerol; AA—arachidonic acid; AEA—*anandamide*; Aβ—beta amyloid; APP—amyloid precursor protein; AMPA-α—Amino-3-hydroxy-5-methyl-4-isoxazolepropionic acid; BDNF—brain-derived neurotrophic factor; CB1R—cannabinoid receptors 1; CB2R—cannabinoid receptors 2; EAE—experimental autoimmune encephalomyelitis; *FGF*-*2*—fibroblast growth factor; GABA—gamma-aminobutyric acid; GDNF—glial cell line-derived neurotrophic factor; IL-1β—interleukin-1beta; IL-6—interleukin 6; IL-10—interleukin 10; LPS—lipopolysaccharide; LTB4—leukotriene B4; (*p-P38*)MAPK—(*phospho*-*P38*) mitogen-activated protein kinase; MCP-1—monocyte chemoattractant protein-1; MMP9—matrix metallopeptidase 9; (p)NF-kB—(phosphorylated) nuclear factor kappa-light-chain-enhancer of activated B cells; NMDA—N-methyl-D-aspartate; OEA—oleoylethanolamide; PEA—palmitoylethanolamide; PGI2—prostaglandin I2; PPARγ—peroxisome proliferator-activated receptor gamma; PSD95—*postsynaptic* density *protein 95*; p-GSK3β—phosphorylated glycogen synthase kinase-3; p25/35—regulatory subunits of cyclin-dependent kinase 5; TNF-α—tumor necrosis factor alpha; TXB2—thromboxane A2; VEGF—vascular endothelial growth factor.

**Table 2:** Preclinical studies assessing MAGL reversible inhibitors utility, conducted between 2015 and 2021.

Therapeutical Application	Compound	Animal Model	Animal Species	Results	Alleged Mechanism	Reference
Pain and inflammatory disorders						
Neuropathic pain	G7 (Figure 9)	Oxaliplatin-induced neuropathic pain	4r5CD-1 mice	Reverse oxaliplatin-induced cold allodynia.	Does not increase 2-AG in brain or spinal cord. Presumably, modulation of 2-AG levels in the peripheral nervous system (not proven)	[47]
Inflammatory pain/other inflammatory disorders	I1 (Figure 11)	Complete Freund’s adjuvant-induced inflammation	Sprague-Dawley rats	Anti-hyperalgesic efficacy correlated with the dose-dependent elevation of brain 2-AG levels.	2-AG increase	[55]
	H5 (Figure 10)	Formalin-induced pain	Wistar rats	Dose-dependent reduction of pain response in both acute and late phases, indicating its peripheral and central effects.	Not mentioned	[53]
Neuropsychiatric disorders						
Depression	G9 (Figure 9)	Reserpine-induced depression	ICR mice	Significantly improved the results of the cumulative immobility time in the forced swim test and tail suspension test induced by reserpine,	Increase in 2-AG	[49]

2-AG—2-arachidonoylglycerol.
